# A Phase I/II Clinical Trial of Pembrolizumab and Cabozantinib in Metastatic Renal Cell Carcinoma

**DOI:** 10.1158/2767-9764.CRC-23-0060

**Published:** 2023-06-08

**Authors:** Elizabeth R. Kessler, Eryn Callihan, Junxiao Hu, Corbin Eule, Geetika Srivastava, Douglas J. Kemme, Praveena Iruku, Vishal Rana, James Moore, Steven R. Schuster, Mali Amirault, Thomas W. Flaig, Elaine T. Lam

**Affiliations:** 1 University of Colorado Cancer Center, University of Colorado Anschutz Medical Campus, Aurora, Colorado.; 2 University of Colorado Cancer Center Biostatistics Core, University of Colorado Anschutz Medical Campus, Aurora, Colorado.; 3 UCHealth Cancer Care and Hematology Clinic, Memorial Hospital Central, Colorado Springs, Colorado.; 4 UCHealth Cancer Center Harmony Campus, Poudre Valley Hospital, Fort Collins, Colorado.; 5 UCHealth Lone Tree Medical Center, Lone Tree, Colorado.

## Abstract

**Purpose:**

Immune checkpoint inhibitor and VEGFR inhibitor combinations are effective treatments for patients with metastatic renal cell carcinoma (mRCC). This phase I/II clinical trial evaluated the safety and efficacy of pembrolizumab and cabozantinib in patients with mRCC.

**Experimental Design:**

Eligible patients had mRCC with clear-cell or non-clear cell histology, adequate organ function, Eastern Cooperative Oncology Group 0–1 performance status, and no prior exposure to pembrolizumab or cabozantinib. The primary endpoint was objective response rate (ORR) at the recommended phase II dose (RP2D). Secondary endpoints included safety, disease control rate (DCR), duration of response (DoR), progression-free survival (PFS), and overall survival (OS).

**Results:**

Forty-five patients were enrolled. A total of 40 patients were treated at the RP2D of pembrolizumab 200 mg i.v. every 3 weeks and cabozantinib 60 mg orally once daily, 38 of which were evaluable for response. The ORR was 65.8% [95% confidence interval (CI), 49.9–78.8] for all evaluable patients [78.6% as first-line therapy, 58.3% as second-line therapy]. The DCR was 97.4% (95% CI, 86.5–99.9). Median DoR was 8.3 months (interquartile range, 4.6–15.1). At a median follow-up of 23.54 months, the median PFS was 10.45 months (95% CI, 6.25–14.63) and median OS was 30.81 months (95% CI, 24.2–not reached). The most common grade 1 and/or 2 treatment-related adverse events (TRAE) were diarrhea, anorexia, dysgeusia, weight loss, and nausea. The most common grade 3 and/or 4 TRAEs were hypertension, hypophosphatemia, alanine transaminase elevation, diarrhea, and fatigue. There was one grade 5 TRAE of reversible posterior encephalopathy syndrome related to cabozantinib.

**Conclusions:**

Pembrolizumab and cabozantinib treatment in patients with mRCC demonstrated encouraging preliminary efficacy and a manageable toxicity profile comparable with other available checkpoint inhibitor-tyrosine kinase inhibitor combinations.

**Trial Registration:**

ClinicalTrials.gov Identifier: NCT03149822 https://clinicaltrials.gov/ct2/show/NCT03149822

**Significance:**

This study evaluated the safety and effectiveness of the combination of pembrolizumab and cabozantinib in patients with mRCC. The safety profile was manageable. The combination showed promising activity with an objective response rate of 65.8%, median PFS of 10.45 months, and median OS of 30.81 months.

## Introduction

Kidney cancer is an important cancer worldwide with an estimated 431,000 new cases globally and 81,800 new cases in the United States with a 5-year survival of 13% for stage IV disease (1, 2). The development of multiple VEGFR tyrosine kinase inhibitors (TKI) and immune checkpoint inhibitors (CPI) has revolutionized the treatment of metastatic renal cell carcinoma (mRCC; refs. 3–9). Sunitinib, a TKI-targeting VEGFR and platelet-derived growth factor receptor, was approved in 2006 and established a new first-line (1 L) standard of care for mRCC (5, 6). The approval of nivolumab, a mAb against the programmed cell death protein 1 (PD-1) cell surface membrane receptor, as a second- or subsequent-line (2 L) treatment in 2015 heralded the CPI era for mRCC (7). Multiple combination therapies are now approved in the front-line setting including nivolumab and ipilimumab, pembrolizumab and axitinib, avelumab and axitinib, nivolumab and cabozantinib, and pembrolizumab and lenvatinib (8–12).

Pembrolizumab, a potent and highly selective humanized monoclonal anti-PD-1 antibody, has demonstrated survival benefit in combination with the VEGFR TKIs axitinib and lenvatinib in the 1 L setting (9, 12). Cabozantinib is an oral, small-molecule TKI that targets MET, VEGFR2/KDR, RET, KIT, FLT3, and AXL that has activity as monotherapy in the 1 L and 2 L settings and in combination with nivolumab in the 1 L setting (7, 11, 13). Preclinical studies have shown multiple mechanisms of synergy between VEGFR and PD-1 inhibition (14–18).

In this phase I/II, open-label study, we evaluated the safety, tolerability, and antitumor activity of pembrolizumab and cabozantinib in patients with mRCC.

## Materials and Methods

### Patient Eligibility

Eligible patients had histologically confirmed, locally advanced, recurrent, or mRCC. Patients with clear-cell RCC (ccRCC) or non-clear cell RCC (nccRCC) were included. Key inclusion criteria were Eastern Cooperative Oncology Group 0–1 performance status, adequate organ function, and resolution of prior treatment-related toxicities to Common Terminology Criteria for Adverse Events (CTCAE) v4.0 grade 1 or less. Key exclusion criteria were prior treatment with pembrolizumab or cabozantinib; prior anticancer mAb therapy within 4 weeks and prior anticancer targeted small-molecule therapy within 2 weeks of day 1 of study treatment; active autoimmune disease requiring systemic treatment in the past 2 years, diagnosis of immunodeficiency, current systemic steroid therapy equivalent to ≥10 mg/day of prednisone; history of osteonecrosis of the jaw, reversible posterior leukoencephalopathy syndrome (RPLS), pneumonitis, or wound dehiscence within 6 months of study; or uncontrolled or significant intercurrent cardiovascular, gastrointestinal, or pulmonary disorders. There was no requirement or limit on the number of prior anticancer therapies.

### Study Design and Treatment

Phase I dose escalation was done using a standard 3 + 3 design. Phase II dose expansion was done using a Simon two-stage design (19–20). Pembrolizumab was administered at a fixed dose of 200 mg by intravenous infusion every 3 weeks each 21-day cycle for up to 35 cycles in the first course. Patients could resume a second course of up to an additional 17 cycles of pembrolizumab treatment if they had disease progression after completing the first course. Cabozantinib was taken orally once daily on a continuous basis at a dose of 40 mg orally once daily or 60 mg orally once daily in phase I, and at the recommended phase II dose (RP2D) in phase II. Cabozantinib dose reductions and interruptions were allowed for management of adverse events (AE). Pembrolizumab was held if needed for AEs. Patients continued treatment in the absence of unacceptable toxicity or clinically compelling disease progression. Treatment beyond progression was allowed if the patient met prespecified criteria and was continuing to derive clinical benefit.

The study was conducted in accordance with the Declaration of Helsinki and Good Clinical Practice Guidelines. The protocol was approved by the Institutional Review Board and all patients provided written informed consent prior to study procedures.

### Study Endpoints

The primary endpoint of the overall study was objective response rate (ORR) in patients treated at the RP2D of pembrolizumab and cabozantinib. Secondary endpoints included dose-limiting toxicities (DLT), MTD, disease control rate (DCR), progression-free survival (PFS), overall survival (OS), duration of treatment, time to response, and duration of response (DoR). Exploratory endpoints included PD-L1 status of archival tumor samples and serum bone turnover markers in patients with bone metastases.

### Antitumor Activity Assessments

Tumor assessments were performed at baseline and every 9 weeks on study. Tumor responses were evaluated based on investigator assessment per RECIST 1.1 (21). ORR was defined as the sum of complete response (CR) and partial response (PR). DCR was defined as the sum of CR + PR + stable disease (SD). Patients with missing or no response assessments were classified as not evaluable. PFS was defined as the time from study treatment initiation to the first occurrence of documented disease progression or death from any cause during the study. OS was defined as the time from study treatment initiation to death from any cause.

### Safety Assessments

DLT was defined as any treatment-related AE (TRAE) grade 3 or higher that occurred during the 21-day DLT assessment window. Any dose-escalation patient who did not complete the DLT period for any reason other than a DLT was replaced by an additional patient at that same dose level. DLT-evaluable patients were required to have received the planned cycle 2 dose of pembrolizumab within 7 days of the planned administration date and to have taken 75% or more of the planned doses of cabozantinib in cycle 1. The MTD was defined as the highest dose level with no more than 1 DLT reported in 6 DLT-evaluable subjects. The RP2D of cabozantinib was selected on the basis of the clinical data, not exceeding the MTD. If <2/6 subjects experience a DLT at 60 mg daily during dose escalation, then 60 mg daily would be considered the RP2D.

AEs were recorded throughout the study and during the follow-up period and graded according to NCI CTCAE Version 4.0 (22). AEs were characterized in terms regarding seriousness, causality, toxicity grading, and action taken regarding trial treatment. Serious AEs (SAE) were defined as any AE that was life threatening; resulted in death, persistent or significant disability/incapacity, hospitalization, or prolongation of an existing inpatient hospitalization; or other important medical events.

### Exploratory Assessments

PD-L1 testing was performed on archival tumor tissues by Discovery Life Sciences (formerly QualTek Molecular Laboratories) using a previously validated IHC assay for PD-L1 using the Merck & Co., Inc proprietary mouse mAb clone 22C3 for the testing of formalin-fixed, paraffin-embedded tissues (23). Interpretation of PD-L1 (22C3) reactivity was performed by evaluating the percentage of tumor, including tumor-infiltrating mononuclear inflammatory cells, which demonstrates membrane staining low (1+), moderate (2+), and high (3+) intensities. The PD-L1 modified percent score (MPS) represents the sum of the percentage of tumor that has low (1+), moderate (2+), and high (3+) intensity. MPS ≥ 1 is considered positive. Bone turnover markers including β C-terminal telopeptide (β-CTX), bone-specific alkaline phosphatase (BS-ALP), and procollagen type 1 amino-terminal propeptide (P1NP) were collected at baseline and on treatment in patients with bone metastases.

### Statistical Analysis

The planned enrollment was estimated to be 6–9 evaluable patients in phase I and 20–38 evaluable patients in phase II. For the primary endpoint of ORR, a Simon two-stage design was used to test the null hypothesis that ORR ≤ 0.20 versus the alternative that ORR ≥ 0.50. After enrollment of 20 evaluable patients in the first stage, the trial would be terminated if 2 or fewer patients respond. If the trial continues to the second stage, a total of 38 patients would be studied. Patients from the phase I dose escalation who were treated at the RP2D were included in the efficacy assessments in Simon stage 1 of the phase II dose expansion.

The ORR, along with a 95% confidence interval (CI), was estimated. The PFS and OS survival curves and medians were estimated using the Kaplan–Meier method and reported with the corresponding two-sided 95% Brookmeyer–Crowley CI, from the on-treatment date to progressive disease (PD), death, or data cut-off date. All statistical analyses were performed by an independent statistician to ensure unbiased data review and conducted on R version 4.1.0, R Core Team (2021).

### Data Availability Statement

Source data generated in this study are not publicly available due to compromise of patient privacy. Derived data supporting the findings of this study may be available from the corresponding author upon request.

## Results

### Patients

**FIGURE 1:**
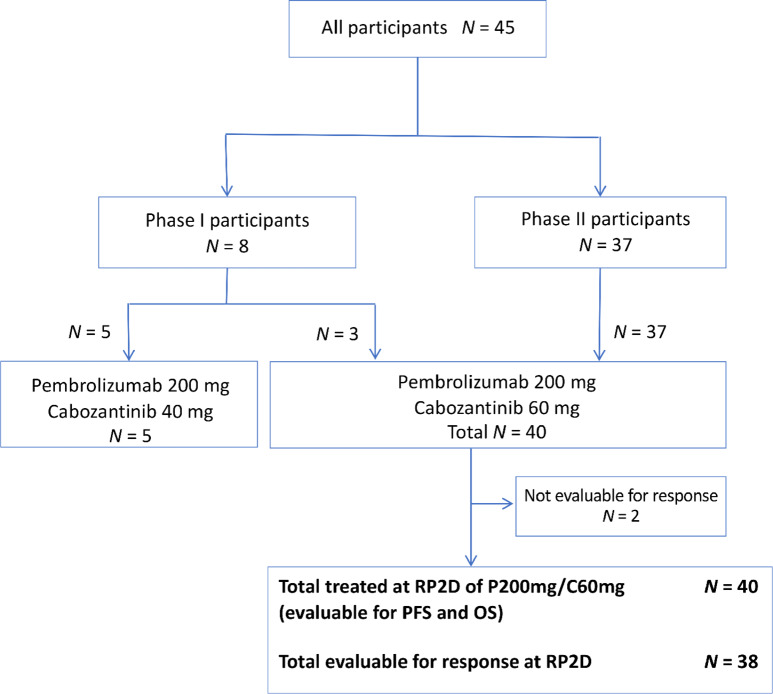
CONSORT diagram and patient disposition. DLT, dose-limiting toxicity; RP2D, recommended phase II dose; P, pembrolizumab; C, cabozantinib.

Between October 2017 and August 2020, 45 patients were enrolled (8 patients in phase I and 37 patients in phase II) across five sites within the University of Colorado/UCHealth system. A total of 40 patients were treated at the RP2D and were evaluable for PFS and OS, and 38 patients were evaluable for response (Fig. 1). Baseline characteristics for all patients are listed in Table 1. Thirty-eight patients (84%) had ccRCC and 7 patients (16%) had nccRCC (5 chromophobe, 2 papillary). Four patients (8.9%) had sarcomatoid differentiation. Thirty patients (67%) had prior exposure to CPI and/or VEGFR therapy. At the time of data analysis, 3 patients remained on study treatment and 42 patients discontinued treatment [26 (62%) due to progression, 14 (33%) due to AEs, and 2 (5%) due to other reasons].

**TABLE 1:** Baseline characteristics

Characteristic	*N* = 45 (%)
Median age, years (IQR)	61.0 (54.0–69.0)
Sex, no. (%)	
Female	12 (26.7)
Male	33 (73.3)
Race, no. (%)	
White	40 (88.9)
Black	3 (6.7)
Asian	2 (4.4)
Ethnicity, no. (%)	
Hispanic	2 (4.4)
Non-Hispanic	38 (84.4)
Unknown/Not reported	5 (11.1)
ECOG, no. (%)	
0	9 (20.0)
1	36 (80.0)
IMDC risk group, no. (%)	
Favorable	6 (13.3)
Intermediate	32 (71.1)
Poor	7 (15.6)
Prior nephrectomy, no. (%)	
Yes	39 (86.7)
No	6 (13.3)
Histology, no. (%)	
Clear cell	38 (84.4)
Non-clear cell	7 (15.6)
Chromophobe	5
Papillary	2
Sites of metastases, no. (%)	
Bone	26 (57.8)
Lung	33 (73.3)
Lymph node	28 (62.2)
Liver	11 (24.4)
Pancreas	4 (8.9)
Other	15 (33.3)
Brain	0 (0)
Line of therapy on study, median (IQR)	2.0 (1.0 to 3.0)
Prior therapy, no. (%)	
VEGFR inhibitor only	10 (22)
Checkpoint inhibitor only	8 (18)
Both VEGFRi and CPI	12 (27)
Neither VEGFRi nor CPI	15 (33)

Abbreviations: CPI, checkpoint inhibitor; ECOG, Eastern Cooperative Oncology Group performance status; IMDC, International Metastatic RCC Database Consortium; IQR, interquartile range; VEGFRi, vascular endothelial growth factor receptor inhibitor.

### MTD and DLT Evaluation

Eight patients were enrolled in phase I [5 patients in the first cohort of pembrolizumab 200 mg and cabozantinib 40 mg (2 were not evaluable for DLT due to missing ≥25% of the planned cabozantinib doses in cycle 1, not related to TRAE) and 3 patients in the second cohort of pembrolizumab 200 mg and cabozantinib 60 mg]. There were no DLTs observed. The MTD and RP2D were determined to be pembrolizumab 200 mg i.v. every 3 weeks and cabozantinib 60 mg orally daily.

### Safety

The most common TRAEs in all 45 patients are reported in Table 2. TRAEs occurred in 44 patients (98%). The five most common grade 1 and 2 AEs were diarrhea (71%), anorexia (62%), dysgeusia (62%), weight loss (62%), and nausea (60%). The most common grade 3 and higher AEs include hypertension (20%), hypophosphatemia (18%), alanine transaminase elevation (11%), diarrhea (9%), and fatigue (7%). Thirty-three patients (73%) experienced a grade 3 AE and there were no grade 4-related AEs. Thirteen patients experienced SAEs, 8 of which were related to treatment: grade 3 transaminitis and hypoglycemia attributed to the combination; grade 3 pancreatitis, nephritis, and pneumonitis attributed to pembrolizumab; and grade 3 pulmonary embolus, confusion due to RPLS, and stroke attributed to cabozantinib. There was one death in the patient with RPLS which was attributed to cabozantinib. TRAEs leading to discontinuation of study treatment occurred in 14 patients (33%): immune-mediated pancreatitis (2), immune-mediated hepatitis (2), intolerable diarrhea (2), elevated liver function tests (LFTs) (2), immune-mediated myocarditis (1), immune-mediated nephritis (1), immune-mediated rash (1), RPLS (1), venous thromboembolism and renal infarcts (1), osteonecrosis of the jaw (1). Twenty-eight of 40 patients (70%) treated at the RP2D required dose reduction of cabozantinib at a median of 4 cycles (range, 1–38).

**TABLE 2:** TRAEs in ≥10% of patients and all grade ≥3

	(*N* = 45) no. (%)	
Adverse event	Grade 1/2	Grade ≥3
Any AE	44 (98)	33 (73)
**Clinical AE**		
Diarrhea	32 (71)	4 (9)
Anorexia	28 (62)	1 (2)
Dysgeusia	28 (62)	0
Weight loss	28 (62)	0
Nausea	27 (60)	0
Palmar-plantar erythrodysesthesia	25 (56)	1 (2)
Fatigue	22 (49)	3 (7)
Mucosal inflammation	19 (42)	2 (4)
Hypertension	17 (38)	9 (20)
Rash	17 (38)	2 (4)
Hair color changes	17 (38)	0
Vomiting	16 (36)	0
Abdominal pain	12 (27)	1 (2)
Arthralgia	12 (27)	0
Dizziness	12 (27)	0
Gastroesophageal reflux	12 (27)	0
Rhinorrhea	11 (24)	0
Dry skin	10 (22)	0
Hoarseness	10 (22)	0
Pruritus	9 (20)	0
Sore throat	9 (20)	0
Constipation	8 (18)	0
Peripheral edema	8 (18)	0
Nasal congestion	8 (18)	0
Headache	7 (16)	0
Dry mouth	7 (16)	0
Epistaxis	6 (13)	0
Dysphagia	6 (13)	0
Dyspnea	5 (11)	0
Generalized muscle weakness	5 (11)	0
Myalgia	5 (11)	0
Dehydration	4 (9)	2 (4)
Oral pain	4 (9)	1 (2)
Cold intolerance	4 (9)	0
Flatulence	4 (9)	0
Hair loss/thinning	4 (9)	0
Muscle cramps	4 (9)	0
Thromboembolic event	2 (4)	1 (2)
Altered mental status	1 (2)	1 (2)
Palpitations	0	3 (7)
Pancreatitis	0	1 (2)
Pneumonitis	1 (2)	1 (2)
RPLS	0	1 (2)^a^
Sepsis	0	1 (2)
**Any laboratory AE**		
AST increased	19 (42)	1 (2)
ALT increased	17 (38)	5 (11)
Hypothyroidism	19 (42)	0
Hypophosphatemia	11 (24)	8 (18)
Proteinuria	12 (27)	0
Anemia	8 (18)	1 (2)
Hypokalemia	8 (18)	0
Increased blood creatinine	5 (11)	2 (4)
Hypomagnesemia	5 (11)	1 (2)
Increased alkaline phosphatase	4 (9)	1 (2)
Hyperglycemia	2 (4)	2 (4)
Hypocalcemia	2 (4)	1 (2)

Abbreviations: AE, adverse event; ALT, alanine transaminase; AST, aspartate transaminase; No., number of patients; RPLS, reversible posterior leukoencephalopathy syndrome.

^a^Grade 5.

### Efficacy

**FIGURE 2:**
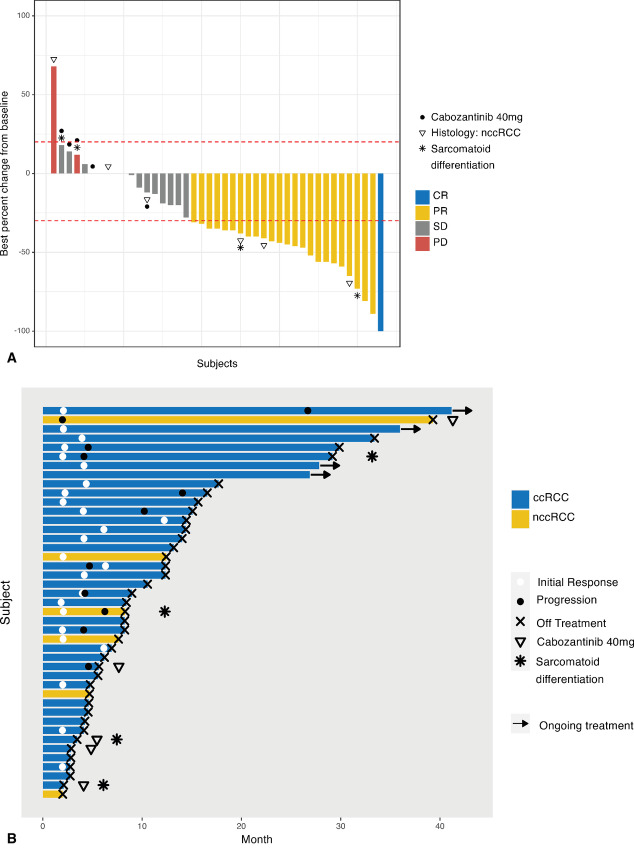
Clinical activity of pembrolizumab and cabozantinib. **A,** Best percent change from baseline as measured by RECIST 1.1 in all evaluable patients from both phase I and phase II (*N* = 43). **B,** Swimmer plot of time on treatment in months for in all evaluable patients from both phase I and phase II (*N* = 43). ccRCC, clear-cell renal cell carcinoma; nccRCC, non-clear cell renal cell carcinoma; CR, complete response; PR, partial response; SD, stable disease; PD, progressive disease.

Thirty-eight patients were evaluable for tumor response at the RP2D (pembrolizumab 200 mg i.v. every 3 weeks and cabozantinib 60 mg orally daily). The ORR was 65.8% (25/38 patients, 95% CI, 49.9–78.8) with 1 CR, 24 PR, 12 SD, 1 PD. The ORR was 78.6% (11/14) in evaluable patients who received study treatment as 1 L therapy and 58.3% (14/24) among patients who received it as second- or subsequent-line therapy. By type or prior therapy, the ORR was 37.5% in patients who had previously received VEGF/R inhibitor only, 62.5% in patients with prior CPI only, 75% in patients who had received both VEGF/R inhibitor and CPI (either in combination or sequentially), and 78.6% in patients who had received no prior therapy (Supplementary Table S1). The ORR was 17% (1/6) in patients without prior nephrectomy and 75% (23/32) in patients with prior nephrectomy. Best response by RECIST in evaluable patients treated at RP2D is presented by histology in Table 3. With the caveat of small number of patients, there were no objective responses among the 5 evaluable patients treated in dose-escalation cohort of pembrolizumab 200 mg and cabozantinib 40 mg (Supplementary Table S2). One patient with chromophobe RCC treated at this dose level had prolonged disease stability lasting over 3 years. The DCR was 97.4% (95% CI, 86.5–99.9). Median DoR was 8.3 months (IQR, 4.6–15.1). The best percentage change in tumor size from baseline and the time on treatment for all evaluable patients in phase I and phase II parts of the study are presented in Fig. 2A and B, respectively. The median duration of treatment was 8.3 months (*n* = 45; IQR, 4.6–15.1), median time to response was 2.2 months (*n* = 25; IQR, 2.0–4.1), and median DoR was 8.3 months (*n* = 25; IQR, 4.0–14.3). Eleven patients (24.4%) were treated beyond progression with a median of 4.9 additional months (range, 1.4–37.8) on treatment.

**TABLE 3:** Best response by RECIST in evaluable patients treated at RP2D (pembrolizumab 200 mg i.v. every 3 weeks and cabozantinib 60 mg orally once daily).

	ccRCC Patients (*N* = 33)	nccRCC Patients (*N* = 5)	All evaluable patients (*N* = 38)
Objective response rate, no. (%, 95% CI)	22 (66.7%, 49.6–80.2)	3 (60%, 23.1–88.2)	25 (65.8%, 49.9–78.8)
Best overall response, no. (%)			
Complete response	1 (3%)	0 (0%)	1 (2.6%)
Partial response	21 (63.6%)	3 (60%)	24 (63.2%)
Stable disease	11 (33.3%)	1 (20%)	12 (31.6%)
Progressive disease	0 (0%)	1 (20%)	1 (2.6%)
Disease control rate, no. (%, 95% CI)	33 (100%, 89.6–100)	4 (80%, 37.6–99)	37 (97.4%, 86.5–99.9)
Time to response, median (range), months	3.1 (1.9–12.4)	2.1 (2.1–2.1)	2.2 (1.9–12.4)

Abbreviations: ccRCC, clear-cell renal cell carcinoma; nccRCC, non-clear cell renal cell carcinoma.

**FIGURE 3:**
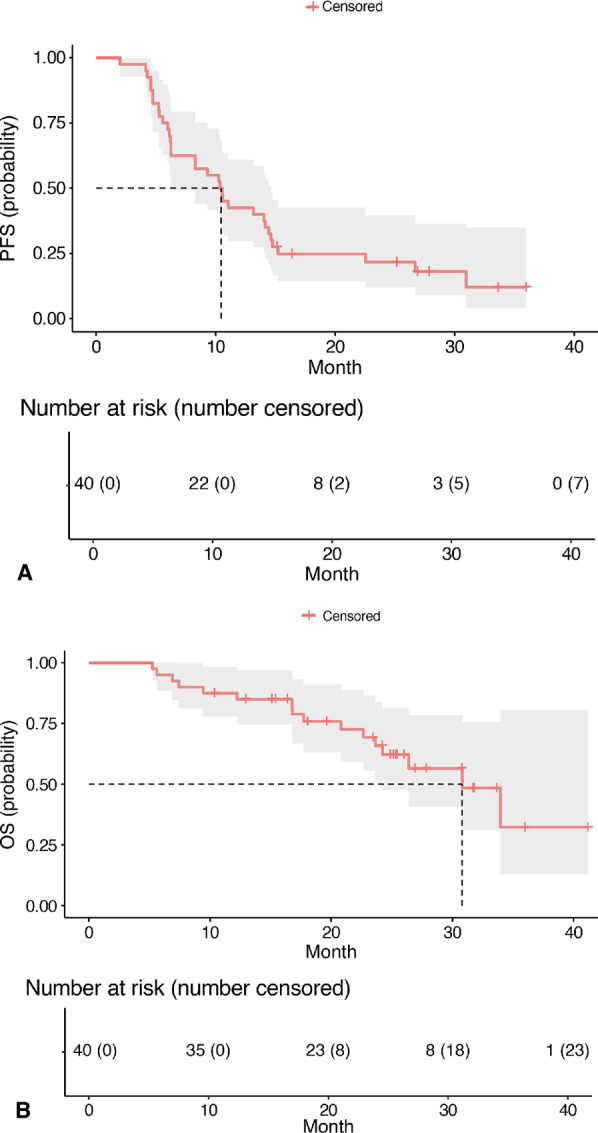
Kaplan–Meier curves of PFS (**A**) and OS (**B**) for all patients treated at the RP2D of pembrolizumab 200 mg i.v. every 3 weeks and cabozantinib 60 mg orally once daily (*N* = 40). Vertical lines show censored patients.

All 40 patients treated at the RP2D were included in the survival analyses. There were 33 PFS events and 16 OS events. The median PFS was 10.45 months (95% CI, 6.25–14.63; Fig. 3A). At a median follow-up of 23.54 months, the median OS was 30.81 months (95% CI, 24.23–not evaluable; Fig. 3B). The median PFS and OS for only patients with ccRCC is presented in Supplementary Fig. S1.

### Exploratory Studies

There were 44 samples of archival tissue available for PD-L1 testing, 40 of which (90.9%) were interpretable and four samples which did not have tumor in the specimen. Of the 40 evaluable samples, 32 (80.0%) had PD-L1 tumor MPS ≥ 1. The ORR was 69% in patients with PD-L1–positive samples and 50% in patients with PD-L1–negative samples. The median MPS of patients with an objective response was 2.5 (range, 0–100) as compared with 10 (range, 0–85) in patients without a treatment response. Two patients with PD had MPS of 20 and 70. The patient with CR had MPS 40.

Twenty-three of 26 patients with bone metastases had bone turnover markers collected at baseline. Very few patients had elevated bone turnover markers at baseline: 1 for beta-cross-linked C-terminal telopeptide (β-CTX; 4.3%), 3 for BS-ALP (13.0%), and 3 for P1NP (13.0%). The ORR in patients with bone metastases was 38.5%. At the time of the first on-treatment scans at 9 weeks, P1NP had decreased in all patients regardless of treatment response. Among patients with PD (2 patients) or a PR (6 patients) who had bone turnover markers collected at baseline and at 9 weeks, change in β-CTX and BS-ALP was concordant with radiographic response in 71.4% and 62.5% patients, respectively.

## Discussion

We conducted this phase I/II study of pembrolizumab and cabozantinib beginning in 2017, prior to the FDA approvals of combination CPI-TKI therapies including pembrolizumab and axitinib in 2019, and nivolumab and cabozantinib and pembrolizumab and lenvatinib in 2021. In our study, the primary endpoint of ORR for all patients treated at the RP2D was met, with ORR 65.8%. The ORR was 73.3% among 1 L patients and 56% among 2 L patients. These results are better than historical results from cabozantinib alone (33% in 1 L and 21% in 2 L+; refs. 7, 13) and similar to the reported ORR for 1 L CPI-TKI studies of axitinib and pembrolizumab (59.3%), cabozantinib and nivolumab (55.7%), lenvatinib and pembrolizumab (71%), as well as the 2 L cabozantinib plus atezolizumab study where ORR was 53% and 58% in the in the 40 mg and 60 mg ccRCC groups, respectively (9, 11, 12, 24). Compared with other landmark trials, our study had a higher proportion of patients with International Metastatic RCC Database Consortium (IMDC) intermediate risk and a lower proportion of patients with IMDC poor risk. In addition, the CR rate of 2.6% in our study is lower compared with landmark 1 L combination studies. One possible explanation for this is that 63% of all evaluable patients (24/38) in our study had prior CPI and/or VEGFR therapy. The ORR in patients with nccRCC was 50%; however, this group represents a small subset of the overall population, and no conclusion can be drawn. Recent studies in patients with nccRCC have shown activity with pembrolizumab monotherapy (ORR 26.7%), cabozantinib monotherapy (ORR 27%), and cabozantinib plus atezolizumab combination (ORR 31%; refs. 24–26). While combination CPI-TKI therapy is well established in the 1 L setting, there are very few studies evaluating combination therapy in the 2 L setting. Our study shows preliminary activity of pembrolizumab and cabozantinib in both the 1 L and 2 L settings for mRCC, suggesting that this combination could be efficacious even after prior CPI and VEGFR exposure.

The safety profile of pembrolizumab plus cabozantinib was manageable and generally consistent with AEs reported with other CPI-TKI combinations. As with the phase I nivolumab and cabozantinib study (27), the AEs related to pembrolizumab and cabozantinib were manageable with early recognition and management of immune-mediated and non–immune-mediated AEs and dose reduction of cabozantinib. While the phase III nivolumab plus cabozantinib combination uses a starting cabozantinib dose of 40 mg/day (28), in our study, the RP2D of cabozantinib in combination with pembrolizumab is 60 mg/day. However, 70% of patients required dose reduction of cabozantinib. This is higher compared with 60% and 46% of cabozantinib monotherapy patients with dose reductions in the METEOR and CABOSUN studies, respectively (7, 13). In general, the incidence of grade 3 or 4 TRAEs with pembrolizumab and cabozantinib in our study was similar to that reported for the cabozantinib plus nivolumab and cabozantinib plus atezolizumab studies (24, 28). As with other CPI-TKI combinations, the overlapping toxicities seen with pembrolizumab plus cabozantinib include diarrhea, elevated liver enzymes, and hypothyroidism. There were no new safety signals in terms of general TRAEs and immune-mediated AEs.

Our exploratory analyses included PD-L1 tumor testing in all patients with archival tissue and bone turnover markers in patients with bone metastases at baseline. In contrast to previously seen responses to pembrolizumab in advanced RCC with positive PD-L1 expression (29), there was no correlation between MPS and ORR in our patients. The degree of MPS positivity also did not correspond with degree of response, as has been demonstrated in non–small cell lung cancer (30). Bone turnover markers were infrequently elevated in patients with bone metastases, indicating their limited diagnostic utility for bone metastases in RCC (31). β-CTX, a marker of bone resorption specific to collagen type 1, most accurately detects PD and PR (32). BS-ALP, a product of osteoblasts, and P1NP, a product of proliferating osteoblasts and fibroblasts, are both markers of bone formation (32). P1NP activity decreased in all patients, making any correlation to treatment possibly coincidental and uninterpretable.

### Limitations

Inherent to small phase I/II studies, the main limitations of this clinical trial include small number of patients, single-arm design with no comparator treatment arm, and absence of randomization. Another limitation is the heterogeneity of the patients included in this study. Because of demonstrated activity of cabozantinib in both ccRCC and nccRCC, we included patients with both histologic subtypes. This may have diluted out the true ORR of this combination in ccRCC tumors alone. However, although numbers of patients with nccRCC were small and no formal conclusions can be made, a response rate of 50% (3/6) gave us insight into potential activity of this CPI-TKI combination in nccRCC. These results are consistent with those of the phase II KEYNOTE-B61 study of 147 untreated patients with metastatic nccRCC which showed promising results of ORR 47.6%, DCR 79.3%, 6-month PFS 72.3%, 6-month OS 87.8% (33).

Our study was initially designed with historical comparisons with single-agent cabozantinib. As cabozantinib monotherapy has approval in both 1 L and ≥2 L settings for mRCC, we also included patients regardless of line of therapy. As ORR is expected to be highest with 1 L and lower with 2 L therapies, combining these cohorts could have overestimated the response in the 2 L setting and underestimated the response in the 1 L setting. What we observed was ORR of 65.8% in all response-evaluable patients, ORR 73.3% (11/15 patients) in 1 L and ORR 56% (14/25 patients) in the 2 L setting, which is similar to the reported ORR with other combinations in 1 L or 2 L settings, and higher when compared with cabozantinib alone in these settings.

## Conclusions

The combination of pembrolizumab and cabozantinib treatment in patients with mRCC demonstrated encouraging preliminary efficacy that is comparable with other available CPI-TKI combinations with a manageable toxicity profile. Further prospective evaluation of pembrolizumab and cabozantinib is warranted, especially in the 2 L treatment setting and in patients with nccRCC.

## Supplementary Material

Supplementary Figure S1 Supplemental Figure S1: Kaplan-Meier curves of (A) progression-free survival (PFS) and (B) overall survival (OS) for patients with clear cell renal cell carcinoma histology (N=34). Vertical lines show censored patients

Supplementary Table S1 Best Response by RECIST in Evaluable Patients Treated at RP2D (Pembrolizumab 200 mg IV Q3W and Cabozantinib 60 mg PO QD) by Prior Therapy

Supplementary Table S2 Best Response by RECIST in Evaluable Patients Treated at the Pembrolizumab 200 mg IV Q3W and Cabozantinib 40 mg PO QD Cohort
