# Supplementary material for: A Phase I/II Clinical Trial of Pembrolizumab and Cabozantinib in Metastatic Renal Cell Carcinoma
Source: Cancer Res Commun. 2023 Jun 8;3(6):1004–12. doi: 10.1158/2767-9764.CRC-23-0060 (PMC10249509; doi:10.1158/2767-9764.CRC-23-0060)
Supplement: Supplementary Figure S1 — Supplemental Figure S1: Kaplan-Meier curves of (A) progression-free survival (PFS) and (B) overall survival (OS) for patients with clear cell renal cell carcinoma histology (N=34). Vertical lines show censored patients [file crc-23-0060-s01.pptx]

## Slide 1
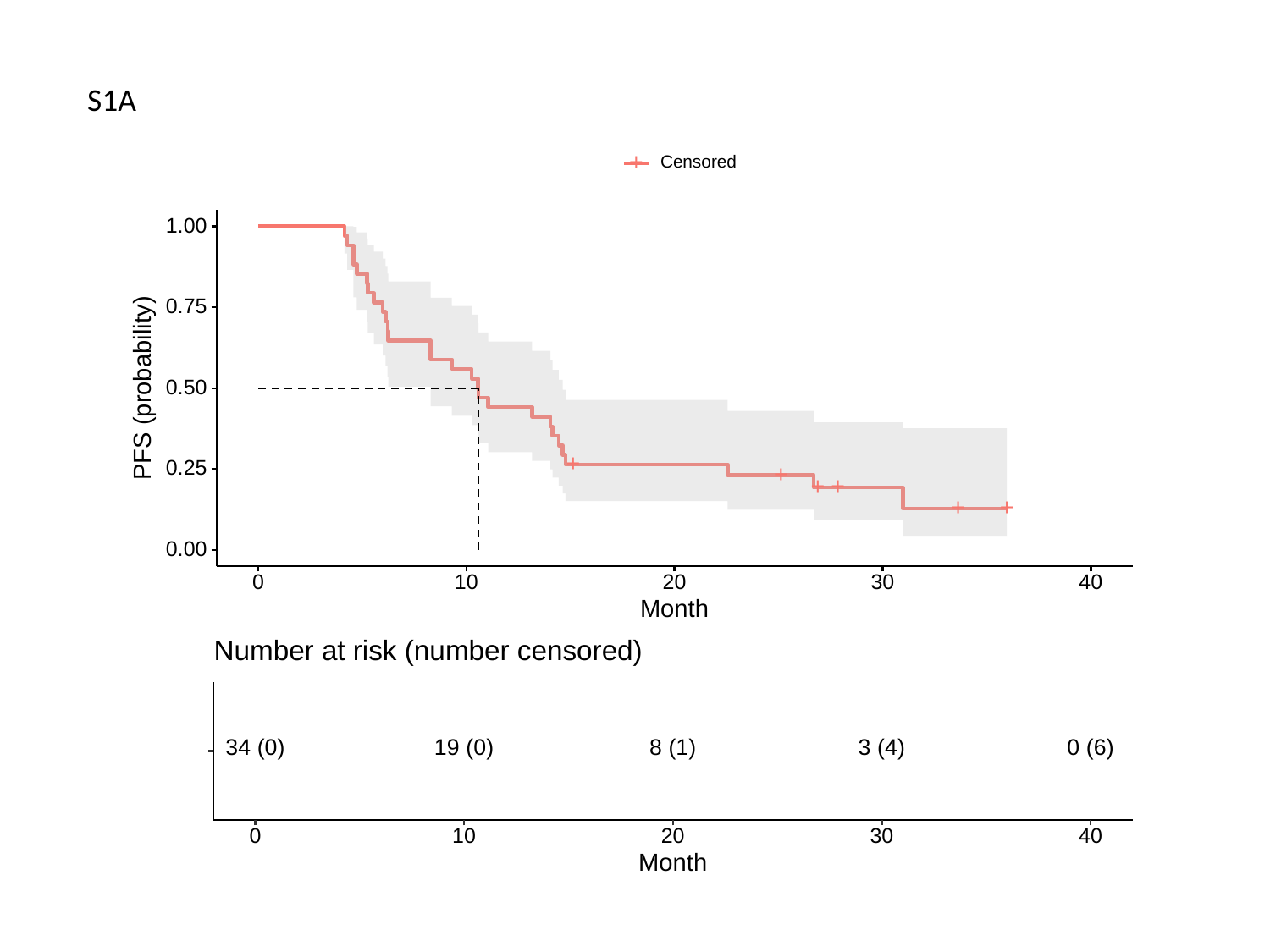

S1A
Censored
+
1.00
0.75
PFS (probability)
0.50
+
0.25
+
+
+
+
+
0.00
20
40
30
0
10
Month
Number at risk (number censored)
34 (0)
19 (0)
8 (1)
3 (4)
0 (6)
20
40
30
0
10
Month

## Slide 2
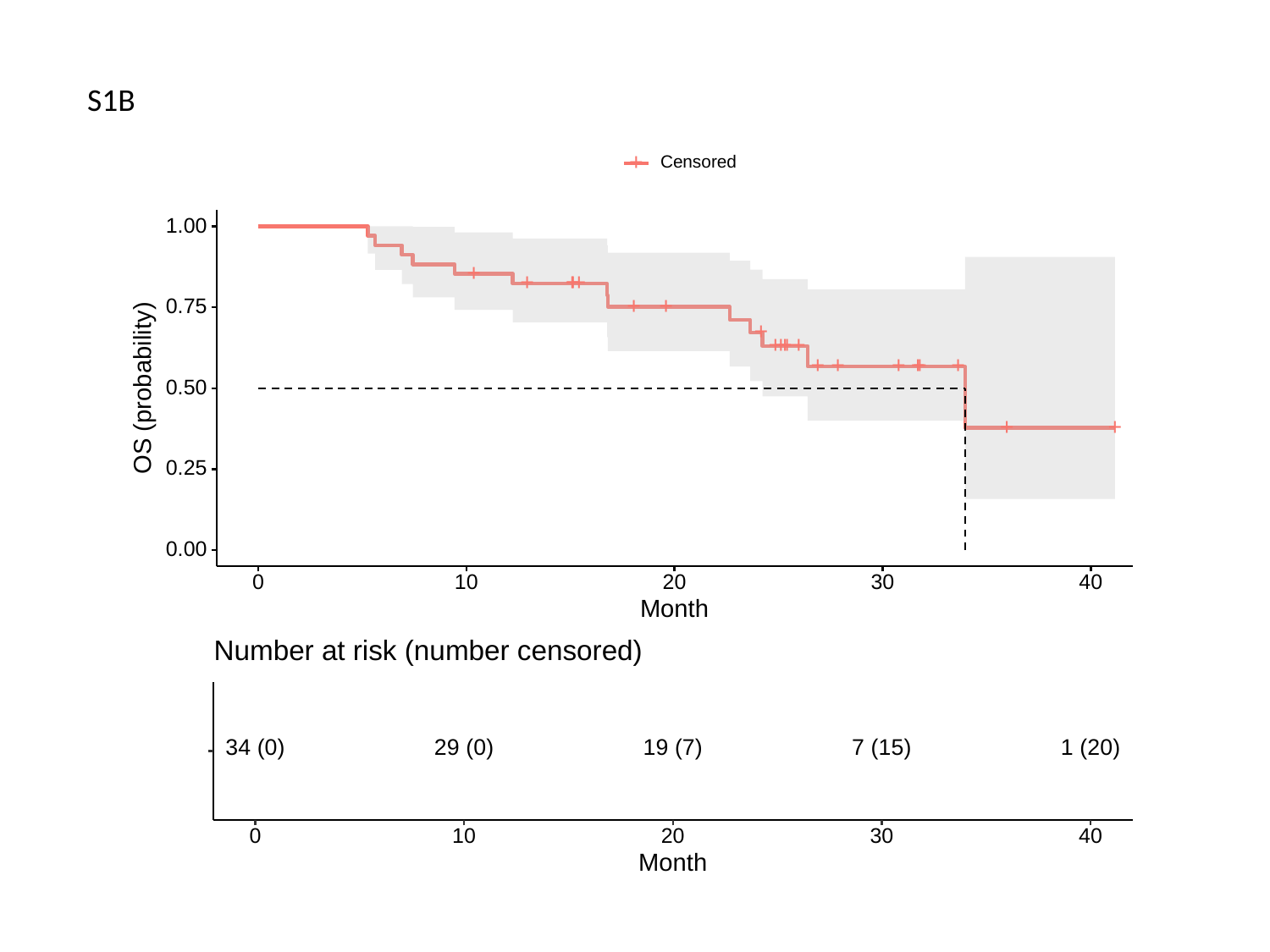

S1B
Censored
+
1.00
+
+
+
+
+
0.75
+
+
+
+
+
+
+
+
+
+
+
+
+
+
OS (probability)
0.50
+
+
0.25
0.00
20
40
30
0
10
Month
Number at risk (number censored)
34 (0)
29 (0)
19 (7)
7 (15)
1 (20)
20
40
30
0
10
Month
