# Supplementary material for: A Phase I/II Clinical Trial of Pembrolizumab and Cabozantinib in Metastatic Renal Cell Carcinoma
Source: Cancer Res Commun. 2023 Jun 8;3(6):1004–12. doi: 10.1158/2767-9764.CRC-23-0060 (PMC10249509; doi:10.1158/2767-9764.CRC-23-0060)
Supplement: Supplementary Table S1 — Best Response by RECIST in Evaluable Patients Treated at RP2D (Pembrolizumab 200 mg IV Q3W and Cabozantinib 60 mg PO QD) by Prior Therapy [file crc-23-0060-s02.docx]

**Supplemental Table S1: Best Response by RECIST in Evaluable Patients Treated at RP2D (Pembrolizumab 200 mg IV Q3W and Cabozantinib 60 mg PO QD) by Prior Therapy**

| **Prior Therapy** | **VEGFRi ONLY**  **(N=8)** | **CPI ONLY**  **(N = 8)** | **VEGFRi and CPI BOTH**  **(N = 8)** | **NEITHER VEGFR or CPI**  **(N = 14)** | **All Patients**  **(N = 38)** |
| --- | --- | --- | --- | --- | --- |
| Objective response rate, No (%) | 3 (37.5%) | 5 (62.5%) | 6 (75%) | 78.6% | 25 (65.8%) |
| Complete response | 0 (0%) | 0 (0%) | 0 (0%) | 1 (7.1%) | 1 (2.6%) |
| Partial response | 3 (37.5%) | 5 (62.5%) | 6 (75%) | 10 (71.4%) | 24 (63.2%) |
| Stable disease | 4 (50%) | 3 (37.5%) | 2 (25%) | 3 (21.4%) | 12 (31.6%) |
| Progressive disease | 1 (12.5%) | 0 (0%) | 0 (0%) | 0 (0%) | 1 (2.6%) |
| Disease control rate,  No (%) | 7 (87.5%) | 8 (100%) | 8 (100%) | 14 (100%) | 37 (97.4%) |

Abbreviations: VEGFRi = vascular endothelial growth factor inhibitor, CPI = checkpoint inhibitor
