# Supplementary material for: A Phase I/II Clinical Trial of Pembrolizumab and Cabozantinib in Metastatic Renal Cell Carcinoma
Source: Cancer Res Commun. 2023 Jun 8;3(6):1004–12. doi: 10.1158/2767-9764.CRC-23-0060 (PMC10249509; doi:10.1158/2767-9764.CRC-23-0060)
Supplement: Supplementary Table S2 — Best Response by RECIST in Evaluable Patients Treated at the Pembrolizumab 200 mg IV Q3W and Cabozantinib 40 mg PO QD Cohort [file crc-23-0060-s03.docx]

**Supplemental Table S2: Best Response by RECIST in Evaluable Patients Treated at the Pembrolizumab 200 mg IV Q3W and Cabozantinib 40 mg PO QD Cohort**

|  | **ccRCC Patients**  **(N=4)** | **nccRCC Patients**  **(N=1)** | **All Evaluable Patients**  **(N=5)** |
| --- | --- | --- | --- |
| Objective response rate, No (%) | 0 (0%) | 0 (0%) | 0 (0%) |
| Best overall response, No (%) |  | | |
| Complete response | 0 (0%) | 0 (0%) | 0 (0%) |
| Partial response | 0 (0%) | 0 (0%) | 0 (0%) |
| Stable disease | 3 (75%) | 1 (100%) | 4 (80%) |
| Progressive disease | 1 (25%) | 0 (0%) | 1 (20%) |
| Disease control rate,  No (%) | 3 (75%) | 1 (100%) | 4 (80%) |

Abbreviations: ccRCC = clear cell renal cell carcinoma, nccRCC = non-clear cell renal cell carcinoma
